# Ezrin Polarization as a Diagnostic Marker for Circulating Tumor Cells in Hepatocellular Carcinoma

**DOI:** 10.3390/cells14010006

**Published:** 2024-12-25

**Authors:** Ibrahim Büdeyri, Olaf Guckelberger, Elsie Oppermann, Dhruvajyoti Roy, Svenja Sliwinski, Felix Becker, Benjamin Struecker, Thomas J. Vogl, Andreas Pascher, Wolf O. Bechstein, Anna Lorentzen, Mathias Heikenwalder, Mazen A. Juratli

**Affiliations:** 1 Department of General, Visceral and Transplant Surgery, University Hospital Muenster, University of Muenster, Albert-Schweitzer-Campus 1, 48149 Muenster, Germany; ibrahim.buedeyri@ukmuenster.de (I.B.);; 2 Department of General, Visceral and Transplant Surgery, Frankfurt University Hospital, 60596 Frankfurt, Germany; 3 Department of Breast Surgical Oncology, The University of Texas MD Anderson Cancer Center, Houston, TX 77054, USA; 4 Department of Diagnostic and Interventional Radiology, Frankfurt University Hospital, Goethe University, 60596 Frankfurt, Germany; 5 Department of Biomedicine, Aarhus University, 8200 Aarhus, Denmark; 6 Division of Chronic Inflammation and Cancer, German Cancer Research Center (DKFZ), 69120 Heidelberg, Germany

**Keywords:** hepatocellular carcinoma, circulating tumor cells, polarization, cancer, single-cell polarity, personalized diagnosis and therapy, liver surgery, ezrin

## Abstract

Hepatocellular carcinoma (HCC) is the sixth most common cancer and the third leading cause of cancer-related death worldwide, with no precise method for early detection. Circulating tumor cells (CTCs) expressing the dynamic polarity of the cytoskeletal membrane protein, ezrin, have been proposed to play a crucial role in tumor progression and metastasis. This study investigated the diagnostic and prognostic potential of polarized circulating tumor cells (p-CTCs) in HCC patients. CTCs were isolated from the peripheral blood of 20 HCC patients and 18 patients with nonmalignant liver disease (NMLD) via an OncoQuick^®^ kit and immunostained with Ezrin-Alexa Fluor 488^®^, CD146-PE, and CD45-APC. A fluorescence microscopy was then performed for analysis. The HCC group exhibited significantly higher levels of p-CTCs, with median values of 0.56 p-CTCs/mL, compared to 0.02 p-CTCs/mL (*p* = 0.03) in the NMLD group. CTCs were detected in 95% of the HCC patients, with a sensitivity of 95% and specificity of 89%. p-CTCs were present in 75% of the HCC patients, with a sensitivity of 75% and a specificity of 94%. Higher p-CTC counts were associated with the significantly longer overall survival in HCC patients (*p* = 0.05). These findings suggest that p-CTCs could serve as valuable diagnostic and prognostic markers for HCC. The incorporation of p-CTCs into diagnostic strategies could enhance therapeutic decision-making and improve patient outcomes.

## 1. Introduction

Hepatocellular carcinoma (HCC) is a prevalent malignant neoplasm with substantial mortality, ranking as the sixth most common cancer and the third leading cause of cancer-related deaths worldwide [1]. The prognosis for HCC remains poor, with an estimated 5-year survival rate of only 12.7% [2,3], largely due to recurrence and metastasis after resection. α-fetoprotein (AFP) is the most commonly used biomarker for the early detection of HCC and the only biomarker validated for clinical use. However, its routine application in HCC surveillance is debated because of issues with specificity, low sensitivity, and limited predictive value [4,5]. The lack of sensitive and precise markers for the early detection of the metastasis and recurrence of liver cancer hampers timely intervention, leading to missed opportunities for effective treatment in many patients. As a result, the 3-year and 5-year survival rates for liver cancer patients remain suboptimal [6]. Therefore, identifying and utilizing novel biomarkers to better monitor the prognosis of HCC patients is crucial. Extensive clinical research is needed to identify therapeutic targets and enhance our understanding of this disease.

Conventional tissue biopsies for HCC research are invasive, whereas liquid biopsy offers a promising noninvasive and real-time approach for analyzing cancer genetics, treatment responses, and therapy resistance [7]. This method assesses circulating components, such as cell-free DNA (cfDNA) [8], cell-free tumor DNA (ctDNA) [9], extracellular vesicles (EVs) [10], tumor-educated blood platelets (TEPs) [11], and circulating tumor cells (CTCs) [12]. Owing to its noninvasive nature, real-time analysis, and ability to capture molecular heterogeneity, liquid biopsy—particularly through CTC analysis—has significant value in precision oncology [13,14]. CTCs, which are shed from primary or metastatic tumors into the bloodstream, are key drivers of metastasis and offer a unique opportunity to study the most aggressive cancer clones, revealing insights into the biology and weaknesses of metastatic spread [15,16,17]. The discovery of CTCs in peripheral blood, first noted in 1869 [18], offers a less invasive diagnostic approach for various cancers [19,20,21,22,23,24]. Despite their low concentration in blood and body fluids, CTCs hold promise as biomarkers for cancer diagnosis, prognosis, early tumor detection, the monitoring of therapeutic responses, and guiding targeted therapies [25,26,27]. Studies on HCC patients have demonstrated their utility in early detection, neoplasm staging, prognostic evaluation and recurrence monitoring. Thus far, numerous studies have investigated CTCs in HCC patients, reporting detection rates varying between 30% and over 80%, depending on the applied methodology and patient population [21,22,23,28,29,30,31,32]. Higher CTC counts often indicate poorer outcomes, including shorter disease-free and overall survival [33,34]. Monitoring changes in CTC levels post-treatment, such as after surgical resection, provides insights into its therapeutic efficacy and recurrence risk [30,35,36,37,38,39].

Ezrin has emerged as a promising alternative biomarker to AFP because of its expression in both AFP-positive [40,41] and AFP-negative HCC [42]. Initially identified in 1983, it belongs to the ezrin/radixin/moesin (ERM) family of proteins and is encoded by the EZR gene [43]. It has various roles in establishing and maintaining cellular polarity and is central to different types of polarization, such as the epithelial polarity or migratory polarity, in mesenchymal cells [44]. Metastasis is marked by polarization plasticity, where tumor cells can undergo various polarization changes by reprogramming their polarity mechanisms through interactions with immune cells or platelets [45,46,47], as well as mechanical entrapment in small vessels [48,49]. During dedifferentiation, tumor cells lose their epithelial characteristics and gain invasive properties through epithelial-to-mesenchymal transition (EMT). This transition enables the cells to become migratory and invade surrounding tissues, as well as intravasate into blood or lymph vessels [50]. CTCs can also display a type of polarity, marked by ezrin-enriched accumulation at one cell end [44]. While the connections between EMT and the polarization of CTCs remain to be formally investigated, it has been hypothesized that both reflect a high polarization plasticity during metastasis. The accumulation of ezrin at one pole creates a “sticky” end, which increases the likelihood of nonspecific interactions with surfaces [44]. This polarized region, upon contact with endothelial cells, brings membrane receptors and ligands close together, facilitating receptor-mediated binding between the tumor cell and the endothelium [44]. In vitro and in vivo assays demonstrated that EMT enhances the metastatic capability of breast and pancreatic carcinoma cells by enabling the interaction of podocalyxin with ezrin, driving cytoskeletal rearrangements essential for initiating cell polarization [51]. An in silico model has suggested that polarized cells attach three times more frequently and exhibit a 1.3-fold increase in adhesion, compared with unpolarized cells, enhancing the chances of extravasation during metastatic spread [50]. In circulation, tumor cells are exposed to immune attacks and blood flow shear stress; thus, their ability to survive, establish at new sites, and initiate metastasis largely relies on successful attachment, adhesion, and extravasation, which help them evade challenging conditions within the bloodstream [16,52]. The reliable detection of CTCs in body fluids remains challenging due to their spatial heterogeneity in the epithelial and mesenchymal makeup [30]. CTCs originating from various tissues differ in size, markers, and immune-phenotyping profiles, complicating detection. Additionally, factors such as cell damage and fragmentation, which can occur either in vivo or during isolation, further limit their clinical applicability [53].

Thus, this study aimed to investigate polarized CTCs (p-CTCs) in HCC patients and compare the findings with those from individuals with nonmalignant liver disease (NMLD).

## 2. Materials and Methods

### 2.1. Study Population and Design

This study was designed as a retrospective cohort study of prospectively collected patient data. We evaluated patients who underwent liver resection at the University Hospital of Frankfurt between 2018 and 2020 and included patients who were willing to provide written informed consent, were older than 18 years of age, and had histopathologically proven HCC. As the control group, patients with NMLD participated in this study. The exclusion criteria were HIV infection and liver metastasis of an extrahepatic origin.

This study included blood samples from 20 patients with HCC and 18 patients with NMLD. Five milliliters of venous blood were drawn from all the patients as a liquid biopsy immediately before resection. The blood was collected in EDTA tubes (Sarstedt, Nümbrecht, Germany).

We examined the demographic, clinical and pathological data of patients with HCC and compared them with those of patients with NMLD. Patient data were extracted from the hospital information system and are shown in detail in Supplementary Table S1. The HCC patients were subsequently monitored for recurrence every six months after resection via computed tomography (CT) or magnetic resonance imaging (MRI) for a median follow-up time of 3.5 years. No follow-up or observational period occurred in the NMLD group.

This study was approved by the ethics committee of the University of Frankfurt on 22 November 2018 (approval number: 321/16). Informed consent forms were signed and obtained from all patients and healthy donors.

### 2.2. Cell Line

HepG2 cells were purchased from CLS (Cell Lines Service GmbH, Eppelheim, Germany) and cultured in DMEM/F-12 supplemented with 10% fetal bovine serum (FBS), 2% HEPES buffer, 1% GlutaMAX, and 1% penicillin/streptomycin (Gibco/Invitrogen, Karlsruhe, Germany). The cells were incubated at 37 °C in a humidified incubator with 5% CO_2_ and were not used after 10 passages.

### 2.3. Antibodies

The following cocktail of fluorescently labeled antibodies was used: (1) anti-CD146-PE (also called Melanoma Cell Adhesion Molecule, MCAM) clone (SHM-57; Biolegend, San Diego, CA, USA) to identify HCC cells; (2) anti-CD45-APC (a known leukocyte antigen) clone (HI30; Biolegend; San Diego, CA, USA) to detect leukocytes; and (3) anti-Ezrin-Alexa Fluor 488 to detect the membrane–cytoskeleton linker protein, ezrin.

Pretrials were performed with HepG2 cell lines and healthy donors to evaluate the sensitivity and specificity of the selected antibodies, as described previously by our research group [54]. The donor blood was divided into 3 × 4 mL samples. Staining was optimized and repeated at least six times. Approximately 200 HepG2 cells were spiked into 2 × 4 mL blood samples. One of the samples served as a negative control and was therefore, not spiked with tumor cells. CTCs were isolated via an OncoQuick^®^ kit (Greiner Bio-One, Frickenhausen, Germany), and staining was performed with 4′,6-diamidino-2-phenylindole (DAPI) (Southern Biotech, USA) to identify nucleated cells. The stained cells were then visualized via widefield fluorescence microscopy, as described in Section 2.6.

Ezrin polarization, as defined in Section 2.5 below, is illustrated in some exemplary HepG2 cells in Supplementary Figures S1 and S2.

### 2.4. Enrichment and Detection of CTCs

**Figure 1:**
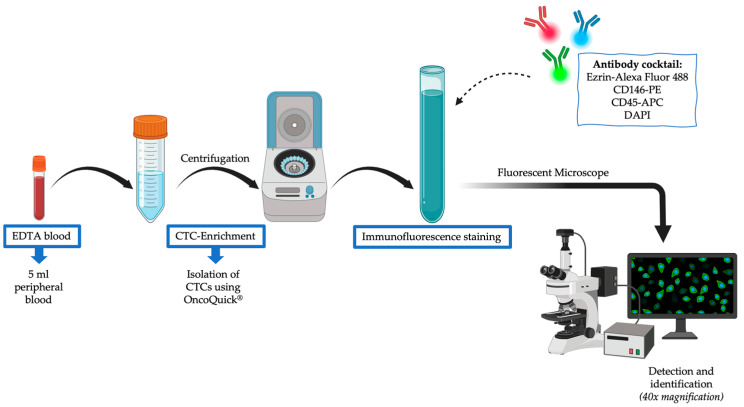
Schematic representation of the workflow. Created in BioRender. Juratli, M. (2023) BioRender.com/k09p009.

After peripheral blood was collected, CTCs were isolated via an OncoQuick^®^ kit no later than 48 h after sampling, as described previously by our research group [54]. Afterwards, the antibody staining of the CTCs was performed using the abovementioned cocktail of fluorescently labeled antibodies, as delineated in Figure 1. Finally, the CTCs were resuspended in FACS buffer, and the samples were analyzed via four-laser FACSAria Fusion (BD Biosciences, CA, USA) and FACS Diva Software (version BD FACSDiva 8.0.1). CTCs were defined as CD45-negative and positive for antibodies against CD146 and ezrin.

### 2.5. Verification via Immunofluorescence Microscopy

In this study, we prepared distinct antibody cocktails, including anti-CD146-PE, anti-CD45-APC and anti-Ezrin-Alexa Fluor 488, to detect a specific subpopulation of CTCs by targeting specific cell surface markers. For that purpose, the antibody staining of the CTCs was performed using the abovementioned cocktail, in addition to DAPI, to stain the nucleated cells. The examples of polarized and unpolarized CTCs are shown in Supplementary Figure S3.

**Figure 2:**
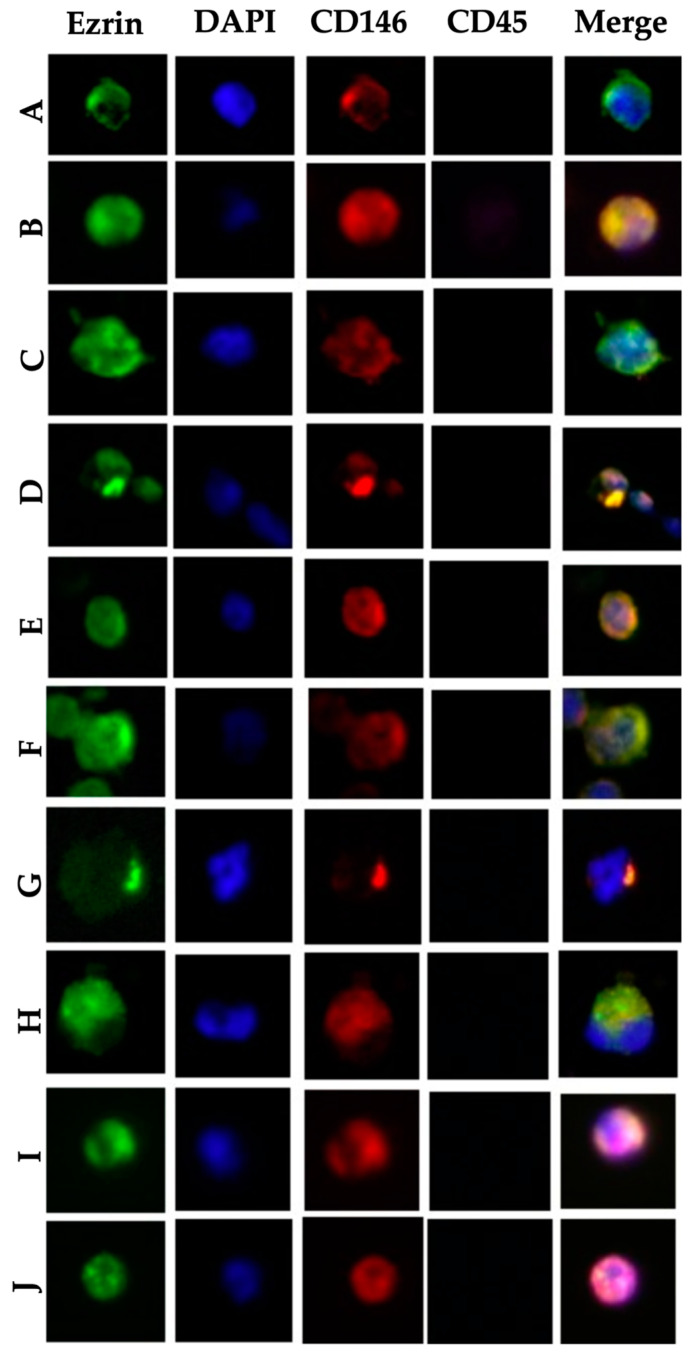
Examples of polarized CTCs from individual patient samples (**A**–**J**). Anti-Ezrin-Alexa Fluor 488 (green), nuclear staining with DAPI (blue), CD146 (red), leukocyte/CD45 (violet) and merged images of all the fluorescence channels. Observed at 40× magnification.

To identify p-CTCs, tumor cells were evaluated via immunofluorescence microscopy, with a focus on the staining patterns, cell morphology, size, shape, and nucleus integrity. First, CTCs were defined based on the following criteria: positive for CD146-PE, negative for CD45-APC, >30 μm in size and an intact nucleus. Among the classified CTCs, we identified p-CTCs as Ezrin-Alexa Fluor 488-positive cells with a visually discernible polarization of ezrin localization. Polarization was defined as one or several localized, subcellular accumulations of twofold or higher fluorescence intensity in the ezrin channel than the average intensity in this channel, comparable to previous descriptions [50]. Visual analyses of all the cells were performed on blinded samples by the same researcher. In Figure 2, the examples of p-CTCs, from 10 individual patient samples, that met the criteria are depicted.

### 2.6. Fluorescence Microscopy

The fluorescence microscopy was performed on a Zeiss Axio Observer Z-1 (Zeiss, Jena, Germany) widefield fluorescence microscope equipped with an Axiocam MRm camera, CCD basic resolution of 1388 × 1040 (1,4 megapixel) and X-Cite Xylis (Excelitas Technologies) broad LED illumination system, with a 40 × 0.75 objective. DAPI was detected using a filter set 49 DAPI shift free (E) EX G 365, BS FT 395, EM BP 445/50. Alexa Fluor 488 was detected using a filter set 52 HE (488 nm) shift free (E) EX BP 488/20, BS FT 505, EM BP 530/50), PE was detected using a filter set 20 Rhodamine shift free (F) EX BP 546/12, BS FT 560, EM BP 575-640, and APC was detected using a filter set 50 Cy 5 shift free (E) EX BP 640/30, BS FT 660, EM BP 690/50. The LED was set at 80% for all channel settings, and the exposure times were set as follows: DAPI for 200 ms, Alexa 488 nm for 588 ms, PE for 350 ms and APC for 720 ms. To ensure reproducibility and reliability, fluorescence thresholds were consistently standardized across all images during analyses. This standardization was applied uniformly to eliminate variability and ensure unbiased quantitative comparisons of fluorescence intensity across different samples.

### 2.7. Statistical Analysis

Continuous variables were assessed for a normal distribution using the Shapiro–Wilk test, histograms, and QQ plots. Normally distributed data are presented as the means (SDs), whereas non-normal or ordinal data are presented as medians (25th; 75th percentiles). Nominal data are presented as N (%). The differences in demographic data between the NMLD and HCC groups were tested using cross tables (X^2^ test) for nominal data and unpaired *t*-tests for continuous data. Correlations were tested using Spearman’s correlation instead of Pearson’s correlation, since most of the variables were ordinal scaled and the continuous variables were not normally distributed. One-way and two-way ANOVAs were used to estimate changes in CTC and p-CTC counts between the NMLD and HCC groups. In the case of assumption violations, bootstrapping (B = 200) was applied. Post hoc group comparisons were made via Yuen tests (effect size: ξ) for a robust ANOVA. ξ = 0.10, ξ = 0.30, and ξ = 0.50 correspond to small, medium, and strong effects, respectively. Recurrence-free survival (RFS) was defined as the time from surgery to recurrence, and overall survival (OS) was defined as the time from surgery to death. Kaplan–Meier analyses and log-rank tests were used to assess survival. Univariate and multivariate Cox regression analyses were applied, with *p* < 0.05 considered as significant. The data analysis was performed using the R statistical software (Version 4.4.0).

## 3. Results

### 3.1. Patient Demographics

The patient demographics are listed in Supplementary Table S1. The median ages in both groups were comparable (68 years in the HCC cohort vs. 67 years in the control cohort, *p* = 0.46). There were more males in the HCC group than in the control group (*n* = 12 vs. *n* = 5, *p* = 0.09). A total of 35% (*n* = 7) of the HCC patients had alcohol abuse in their medical history. Additionally, 25% (*n* = 5) of the participants had metabolic dysfunction-associated steatohepatitis (MASH), and 25% (*n* = 5) were diagnosed with type 2 diabetes mellitus. A total of 55% of the HCC patients (*n* = 11) presented with liver cirrhosis, and 35% (*n* = 7) presented with metastatic disease, at the time of diagnosis. Half of the HCC patients were AFP-positive (>7 ng/mL).

HCC patients had varying MELD scores (0–10: 17, 11–20: 2, 21–30: 0, 31–40: 1). Most patients were classified as BCLC stage A (*n* = 7, 35%), followed by stage C (*n* = 6, 30%), stage B (*n* = 5, 25%) and stage 0 (*n* = 2, 10%). The median tumor size was 3.8 cm, ranging from 0.7–14 cm, with 75% of the tumors measuring less than 5 cm. A total of 18 out of 20 HCC patients underwent resection, with 78% undergoing open surgery rather than laparoscopic surgery. Preoperative therapy, either microwave ablation (MWA) or transarterial chemoembolization (TACE), was administered to 25% of the patients. Only 17% of resected HCC patients had high-grade tumors (grade 3), 22% showed microvascular invasion (V1), and 89% achieved tumor-free resection margins (R0). The recurrence rate was 17%. The mortality rates were comparable between the HCC (15%) and NMLD (16.7%) groups during the study period.

The HCC group presented significantly higher levels of CTCs and p-CTCs, with median values of 1.59 CTCs/mL (Table 1) and 0.56 p-CTCs/mL (Table 2), respectively, compared with 0.05 CTCs/mL (*p* = 0.001) and 0.02 p-CTCs/mL (*p* = 0.03) in the NMLD group, respectively.

**Figure 3:**
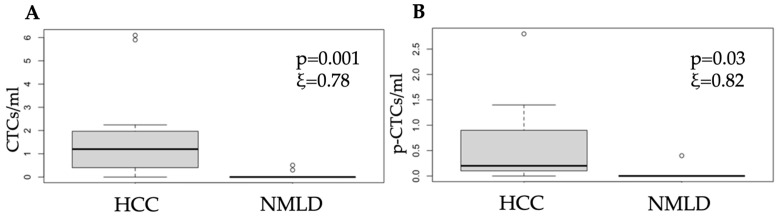
Boxplots comparing (**A**) CTCs and (**B**) p-CTCs between patients with HCC and those with NMLD. The lines within each box represent the median values, the boxes’ limits indicate the first and third quartiles, and the whiskers represent the smallest and largest values within 1.5 times of the IQRs from the first and third quartiles. *p*-values and ξ-effect sizes were determined using one-way ANOVA, and *p* < 0.05 was considered significant.

The representative boxplots can be visualized in Figure 3.

### 3.2. Sensitivity and Specificity Analysis of CTCs and P-CTCs

As demonstrated in Table 3, CTCs were detected in 19 HCC patients (95%). The false positivity rate was 11% (*n* = 2, 95% CI: 1%, 35%), with a sensitivity of 95% (95% CI: 75%, 100%) and specificity of 89% (95% CI: 65%, 99%). The positive predictive value was 90% (95% CI: 70%, 99%), and the negative predictive value was 94% (95% CI: 71%, 100%).

As shown in Table 4, p-CTCs were found in 15 HCC patients (75%). The false positivity rate was 6% (*n* = 1, 95% CI: 0%, 27%), with a sensitivity of 75% (95% CI: 51%, 91%) and specificity of 94% (95% CI: 73%, 100%). The positive predictive value was 94% (95% CI: 70%, 100%), and the negative predictive value was 77% (95% CI: 55%, 92%).

### 3.3. Factors Correlated with CTCs and P-CTCs

The number of CTCs and p-CTCs in the peripheral blood was positively correlated with the malignant nature of the disease, i.e., HCC (*rs* = 0.82, *p* < 0.001 and *rs* = 0.67, *p* < 0.001, respectively), as opposed to NMLD. Among the HCC patients, a negative correlation was found between the AFP level (*rs* = −0.21, *p* = 0.36), tumor grade (*rs* = −0.51, *p* = 0.03), tumor size >5 cm (*rs* = −0.53, *p* = 0.02), age greater than 70 years (*rs* = −0.45, *p* = 0.047) and the number of p-CTCs.

Additionally, there was a negative correlation between the AFP level (*rs* = −0.23, *p* = 0.32), tumor grade (*rs* = −0.46, *p* = 0.06) and the number of CTCs in the HCC group. Interestingly, a tumor size > 5 cm was positively correlated with the number of CTCs among HCC patients (*rs* = 0.05, *p* = 0.8).

### 3.4. Analysis of Variance Comparing Means Across the Variables in the HCC Cohort

Patients with a tumor size > 5 cm tended to have more CTCs/mL than those with a smaller tumor (M = 1.68, SD = 0.70 vs. M = 1.10, SD = 1.91; *p* = 0.19, ξ = 0.45 [0.00; 0.95]) but significantly fewer p-CTCs/mL (M = 0.07, SD = 0.11 vs. M = 0.58, SD = 0.74, respectively; *p* = 0.04, ξ = 0.76 [0.00; 0.99]). Interestingly, patients older than 70 years tended to have more CTCs/mL than those younger than 70 years (M = 1.26, SD = 0.8 vs. M = 1.22, SD = 1.95, respectively, *p* = 0.93, ξ = 0.17 [0.00; 0.89]) but significantly fewer p-CTCs/mL (M = 0.1, SD = 0.23 vs. M = 0.6, SD = 0.77, respectively, *p* = 0.02, ξ = 0.67 [0.00; 0.97]). Those with high-grade tumors (grade 3) tended to have fewer CTCs/mL than those with lower-grade tumors (grades 1 and 2) (M = 0.87, SD = 0.81 vs. M = 1.51, SD = 1.82; *p* = 0.38, ξ = 0.55 [0.00; 0.79]) and fewer p-CTCs/mL (M = 0.13, SD = 0.11 vs. M = 0.58, SD = 0.75, respectively, *p* = 0.06, ξ = 0.30 [0.00; 1.00]). In parallel, patients with low-grade tumors (Grade 1) tended to have more CTCs/mL than those with high-grade tumors (Grade 2 and 3) (M = 4.1, SD = 2.82 vs. M = 1.19, SD = 1.41, respectively, *p* = 0.31, ξ = 0.34 [0.00; 1.00]) and significantly more p-CTCs/mL (M = 1.3, SD = 0.14 vs. M = 0.34, SD = 0.72, respectively, *p* = 0.001, ξ = 0.69 [0.04; 0.79]). In addition, patients with viral hepatitis tended to have more CTCs/mL than those without viral hepatitis (M = 3,27, SD = 2.28 vs. M = 1.01, SD = 1.44, respectively, *p* = 0.23, ξ = 0.65 [0.00; 0.99]) and more p-CTCs/mL (M = 1.67, SD = 0.99 vs. M = 0.25, SD = 0.44, respectively, *p* = 0.12, ξ = 0.89 [0.00; 1.00]). Finally, patients with previous treatments tended to have more CTCs/mL than patients without previous treatments (M = 2.87, SD = 2.80 vs. M = 1.12, SD = 0.79, respectively, *p* = 0.51, ξ = 0.40 [0.00; 0.97]) and significantly more p-CTCs/mL (M = 1.27, SD = 0.99 vs. M = 0.22, SD = 0.33, respectively, *p* < 0.01, ξ = 0.90 [0.07; 0.98]).

### 3.5. Overall and Recurrence-Free Survival Probabilities in the HCC Cohort

We used Kaplan–Meier survival curves with the log-rank test to determine the probabilities of overall survival (OS) and recurrence-free survival (RFS) among HCC patients with and without p-CTCs.

**Figure 4:**
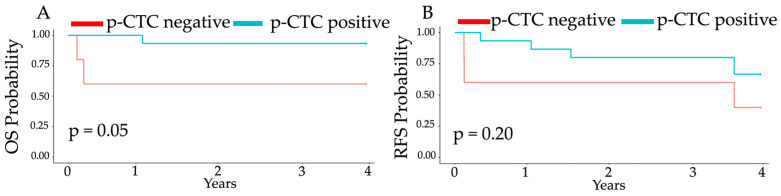
Kaplan–Meier analysis of HCC patients with and without p-CTCs. (**A)** Overall survival (OS) curve and (**B)** recurrence-free survival (RFS) curve. HCC patients with p-CTCs exhibited a longer mean overall survival (40 ± 8 months vs. 26 ± 22 months, *p* = 0.05). However, the difference in mean recurrence-free survival between HCC patients with and without p-CTCs was not statistically significant (36 ± 13 months vs. 25 ± 22 months, respectively; *p* = 0.20).

As depicted in Figure 4, there was a significant difference in the overall survival probability across HCC patients with or without p-CTCs (*p* = 0.05), although there was no statistically significant difference in the recurrence-free survival probability across HCC patients with or without p-CTCs (*p* = 0.20) within the follow-up period.

### 3.6. Univariate Cox Proportional Hazards Analysis for Overall Survival

**Figure 5:**
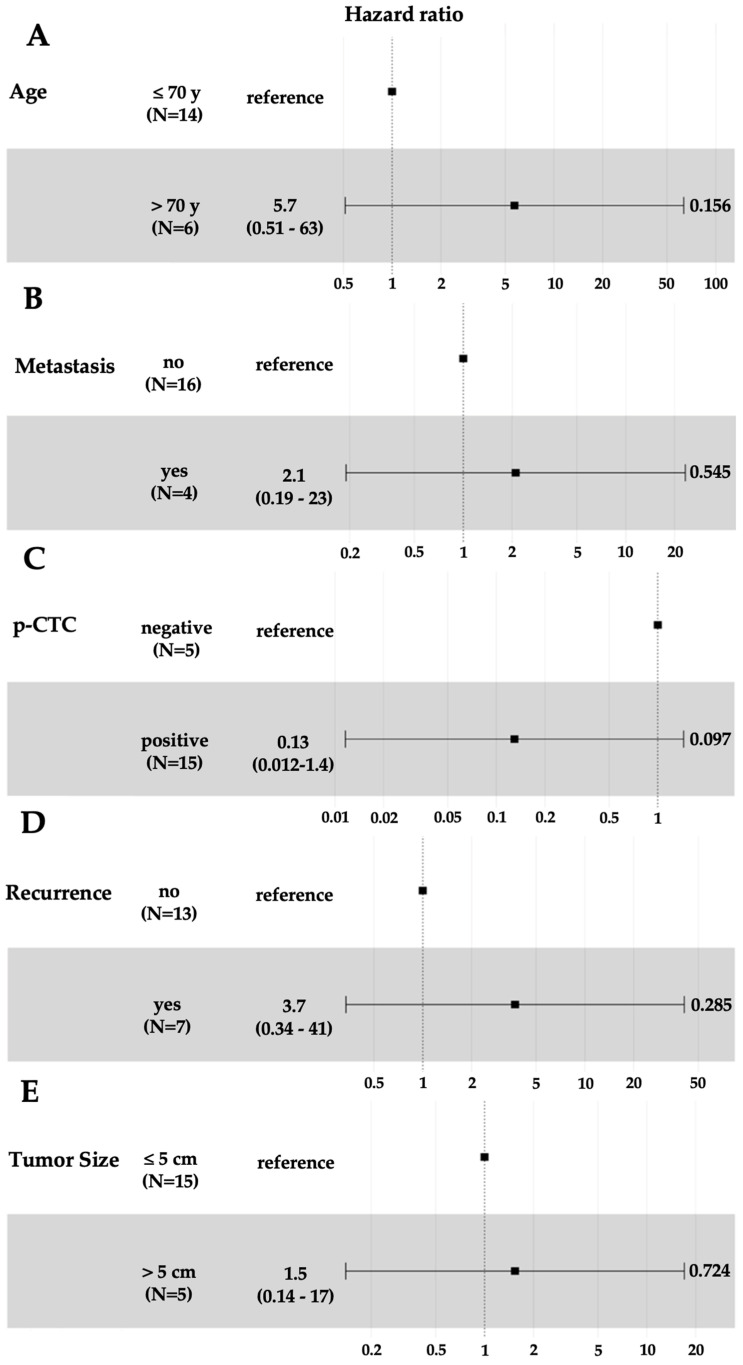
Forest plot of hazard ratios for age > 70 years (**A**), metastasis (**B**), p-CTC (**C**), recurrence (**D**) and tumor size >5 cm (**E**) in HCC patients. Hazard ratios were calculated via univariate Cox regression analysis.

In the univariate Cox regression analysis of overall survival shown in Figure 5, we did not observe any significant difference in the risk of death for HCC patients with respect to those older than 70 years (HR = 5.7; 95% CI 0.51–63, *p* = 0.16), metastasis (HR = 2.1; 95% CI 0.19–23, *p* = 0.54), recurrence (HR = 3.7; 95% CI 0.34–41, *p* = 0.28) or tumor size >5 cm (HR = 1.5; 95% CI 0.14–17, *p* = 0.72). However, again, there was no significant difference in the risk of death for HCC patients with p-CTCs compared with those without p-CTCs (HR = 0.13; 95% CI 0.012–1.4, *p* = 0.1). Owing to the small sample size and the lack of statistical significance in the univariate analysis, no significant results could be delivered through the multivariate analysis.

## 4. Discussion

This study aimed to evaluate the diagnostic and prognostic potential of the localization of the cytoskeletal membrane protein ezrin within CTCs in HCC patients. Our research group utilized an optimized methodology to isolate and identify CTCs from peripheral blood. While previous studies detected ezrin expression in tumor tissues, Zhong et al. first reported ezrin, in 2017, in CTCs from osteosarcoma patients at the gene level [55]. Lorentzen et al. later described the polarization of ezrin in CTCs from breast, pancreatic, and lung cancer patients [50]. Our study is the first to demonstrate ezrin polarization in the CTCs of HCC patients and its association with overall survival.

Ezrin-mediated cell polarity plays an essential role in tumor cell adhesion and the ability of tumor cells to move across the endothelium. Ezrin directly affects metastasis by regulating cell shape, adhesion, and movement through remodeling the actin cytoskeleton [56]. Peng et al. [57] described this process in mouse models, whereas Etienne-Manneville et al. [58] were the first to propose a connection between cell polarity and migration. In a mouse model, Chen et al. demonstrated that the phosphorylation of ezrin at the Thr567 residue significantly increased HCC invasiveness and its likelihood of metastasis [59]. Liu et al. reported that inhibiting TRPV4 in a mouse model altered the degree of single-cell polarization and significantly reduced the metastatic potential of HCC cells by impairing their adhesion to vascular endothelial cells [60]. A recent meta-analysis by Liang et al. investigated the prognostic significance of ezrin in gastrointestinal and colorectal cancers and revealed that ezrin expression was significantly linked to tumor stage and lymph node status [61]. In a study involving 104 HCC patients, Yeh et al. concluded that ezrin overexpression was associated with smaller tumor sizes, poorer differentiation, and increased vascular invasion [41]. Additionally, ezrin-positive patients had higher levels of serum α-fetoprotein, shorter recurrence-free survival, and lower overall survival rates [40]. In a pancreatic cell line, Meng et al. reported the increased motility in pancreatic cancer cells with ezrin overexpression [62], whereas Lin et al. reported a negative correlation between ezrin levels and metastasis in colorectal cancer [63]. Lin et al. also reported that the 5-year survival rate for patients with low ezrin expression was significantly lower and concluded that the absence of ezrin is linked to a poor prognosis. Hiscox and Jiang reached a similar conclusion in their investigations of colon cancer cell lines [64]. The role of increased ezrin in cancer metastasis varies between different cancer types. However, the relevance of these findings to the expression of ezrin in CTCs, particularly regarding its localization, remains limited.

CTC detection rates in HCC patients vary across studies, ranging from 40% [65] to 100% [66], depending on the methodology. Guo et al. [67] highlighted the prognostic significance of CTCs in predicting tumor recurrence, with higher recurrence rates in patients with higher CTC loads. A meta-analysis by Fan et al. confirmed the link between the presence of CTCs and a poor prognosis in patients with HCC [68]. However, Sun et al. [29] argued that the numerical collection of CTCs alone offers a limited diagnostic value. Our findings demonstrated that HCC patients had significantly greater numbers of CTCs and p-CTCs than NMLD patients. Interestingly, two patients in the NMLD group also had detectable CTCs, likely due to the epithelial mesenchymal transition (EMT) related to liver inflammation.

A correlational analysis revealed that the number of p-CTCs was negatively correlated with tumor grade, size, and age over 70 years. HCC patients younger than 70 years with a tumor size ≤5 cm and low-grade tumors presented higher p-CTC counts. One explanation for this phenomenon could be that p-CTCs enter the bloodstream at a relatively early stage, specifically at the onset of tumorigenesis [69,70,71,72]. The parallel progression model suggests that tumor growth and metastasis can progress simultaneously [71]. According to this model, the metastasis process does not necessarily lag behind tumor formation. Smaller tumors may have a greater activity in terms of p-CTC release. These tumors might have better access to the vascular system due to their enhanced blood supply, thus facilitating the release of p-CTCs. Another important consideration is tumor heterogeneity, which could play a significant role in these findings. Tumors often consist of diverse cell populations with varying degrees of aggressiveness, differentiation, and metastatic potential [73]. This heterogeneity could affect the characteristics of p-CTCs released into the bloodstream. Notably, patients who received preoperative therapies had higher p-CTC counts, potentially because immune suppression facilitates tumor cell mobilization [74,75,76]. While we found no independent prognostic factors in our multivariate analysis, higher p-CTC counts were associated with a significantly longer overall survival in HCC patients. These findings suggest that p-CTCs may have prognostic value in this patient population. While earlier studies have shown a direct correlation between polarization and metastatic capacity in vitro [50], we demonstrated that this concept might be far more complex in HCC patients. The role of ezrin in CTC behavior and prognosis remains controversial. The prognostic significance of ezrin expression in CTCs, particularly in HCC, warrants further investigation.

This retrospective study has inherent limitations, including the potential selection bias due to its reliance on a tertiary center cohort and the relatively small sample size of 20 HCC patients and 18 NMLD patients. These limitations restrict the generalizability of our findings and emphasize the preliminary nature of the results. To strengthen the causal link between ezrin polarization and metastatic potential, larger, blinded, and prospective studies involving more diverse patient populations are needed. Such future studies will help validate the clinical relevance of these findings and determine the applicability of p-CTCs as prognostic biomarkers in HCC.

This study has certain limitations that must be acknowledged. While our data strongly suggest that the observed cells are CTCs based on their marker profile (Ezrin+, CD146+, CD45−, DAPI+), we acknowledge the possibility that some of these cells could represent circulating endothelial cells (CECs), as CD146 is also expressed in endothelial populations. However, the absence of CD45 expression, along with the presence of ezrin—a marker more commonly associated with tumor cells—strongly supports the hypothesis that these cells are indeed CTCs. Despite this, further molecular characterization and functional validation are necessary to definitively confirm their identity. Being a retrospective analysis conducted within a tertiary center cohort, there is an inherent risk of selection bias. Additionally, the relatively small sample size, comprising 20 HCC patients and 18 NMLD patients, limits the statistical robustness and generalizability of our findings to broader patient populations. These limitations emphasize the preliminary nature of our results and the need for further investigation.

To establish a more definitive link between ezrin polarization and metastatic potential, future studies should incorporate larger, multicenter, blinded, and prospective cohorts with diverse patient populations. Such studies would not only enhance the validity of our findings but also clarify the clinical utility of p-CTCs as prognostic biomarkers in HCC. Ultimately, this approach could help bridge the gap between experimental evidence and clinical application, advancing the field of liquid biopsy-based prognostics in hepatocellular carcinoma.

## 5. Conclusions

Our study suggests that CTCs may hold potential as biomarkers for early tumor progression in HCC. However, these findings are preliminary, and further validation through larger, prospective studies to confirm the diagnostic and prognostic utility of p-CTCs in clinical settings is needed. Considering potential confounding factors, such as disease stage and treatment history—including prior chemotherapy or local ablative treatments—is crucial, as these factors could affect ezrin polarization in CTCs and, subsequently, influence the study’s results. Definitive conclusions regarding their clinical applicability should be approached with caution until more robust data from well-designed trials become available. Future research should focus on refining detection methodologies, addressing potential confounding factors, and evaluating p-CTCs in diverse clinical populations to strengthen their translational potential. Additionally, an in-depth investigation of the molecular characteristics of p-CTCs may help determine whether they could serve as viable therapeutic targets to mitigate the risk of recurrence and metastasis. Techniques such as PCR-based detection [77] or the analysis of extracellular vesicle surface protein expression [78] may further enhance detection capabilities in future studies. Overall, while the findings are promising, cautious interpretation is warranted, given the study’s inherent limitations. 

## Figures and Tables

**Table 1:** Descriptive statistics of the number of CTCs per mL blood sample. M and SD represent the mean and standard deviation, respectively. LL and UL indicate the lower and upper limits of the 95% confidence interval for the mean, respectively.

Cohort	M	M 95% CI [LL, UL]	SD
HCC	1.59	[0.81, 2.38]	1.68
NMLD	0.05	[−0.02, 0.11]	0.14

**Table 2:** Descriptive statistics of p-CTCs per mL blood sample. M and SD represent the mean and standard deviation, respectively. LL and UL indicate the lower and upper limits of the 95% confidence interval for the mean, respectively.

Cohort	M	M 95% CI [LL, UL]	SD
HCC	0.56	[0.23, 0.89]	0.70
NMLD	0.02	[−0.02, 0.07]	0.09

**Table 3:** Confusion matrix for the presence of CTCs. The accuracy of our method is 92%, and the precision is 90%.

	Patients with HCC	Patients with NMLD	Sum
Positive	19 (95%)	2 (11%)	Σ = 21
Negative	1 (5%)	16 (88.9%)	Σ = 17
sum	20	18	Σ = 38

**Table 4:** Confusion matrix for the presence of p-CTCs. The accuracy of our method is 84%, and the precision is 94%.

	Patients with HCC	Patients with NMLD	Sum
Positive	15 (75%)	1 (5.6%)	Σ = 16
Negative	5 (25%)	17 (94.4%)	Σ = 22
sum	20	18	Σ = 38

## Data Availability

The datasets used and analyzed during the present study are available from the corresponding author upon reasonable request.

## Supplementary Materials

The following supporting information can be downloaded at https://www.mdpi.com/article/10.3390/cells14010006/s1, Figure S1: HepG2 tumor cell line with anti-Ezrin-Alexa Fluor 488 staining, Figure S2: Example of polarized and unpolarized HepG2 cells, Figure S3: Anti-Ezrin-Alexa Fluor 488 staining of polarized and unpolarized tumor cells. Table S1: Demographics, Table S2: Means, standard deviations and correlations with confidence intervals in the HCC group.
