# Supplementary material for: Ezrin Polarization as a Diagnostic Marker for Circulating Tumor Cells in Hepatocellular Carcinoma
Source: Cells. 2024 Dec 25;14(1):6. doi: 10.3390/cells14010006 (PMC11720075; doi:10.3390/cells14010006)
Supplement: Supplementary file 1 [file cells-14-00006-s001.zip › cells-3308875-supplementary.pdf]

Supplementary Materials: The following supporting information can be downloaded at: www.mdpi.com/xxx/s1, Figure S1: HepG2 tumor cell line with anti-Ezrin-Alexa Fluor 488 staining, Figure S2: Example of polarized and unpolarized HepG2 cells, Figure S3: Anti-Ezrin-Alexa Fluor 488 staining of polarized and unpolarized tumor cells. Table S1: Demographics, Table S2: Means, standard deviations and correlations with confidence intervals in the HCC group.

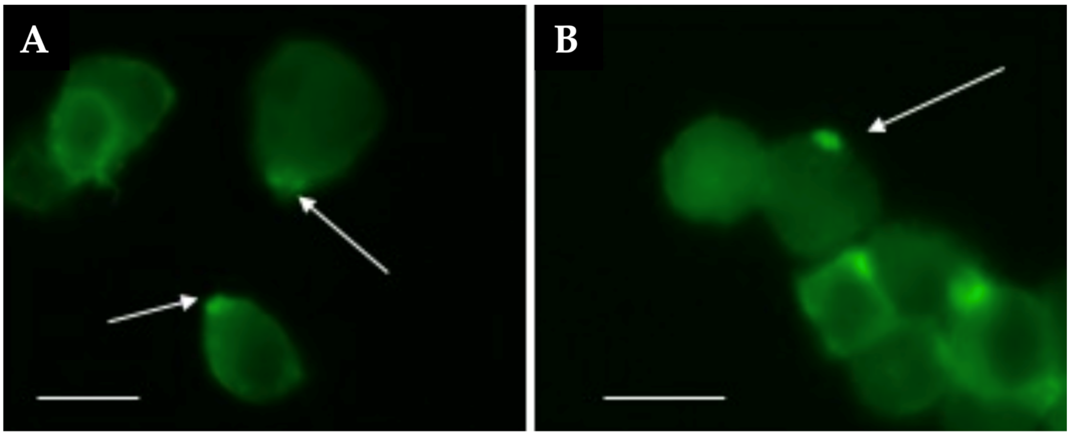

Supplementary Figure S1. HepG2 tumor cell line with anti-Ezrin-Alexa Fluor 488 staining in **A)** and **B)**. The white arrows point to the polarization of Ezrin at the apical pole of the cells. Scale bar = 50 µm. 40x magnification.

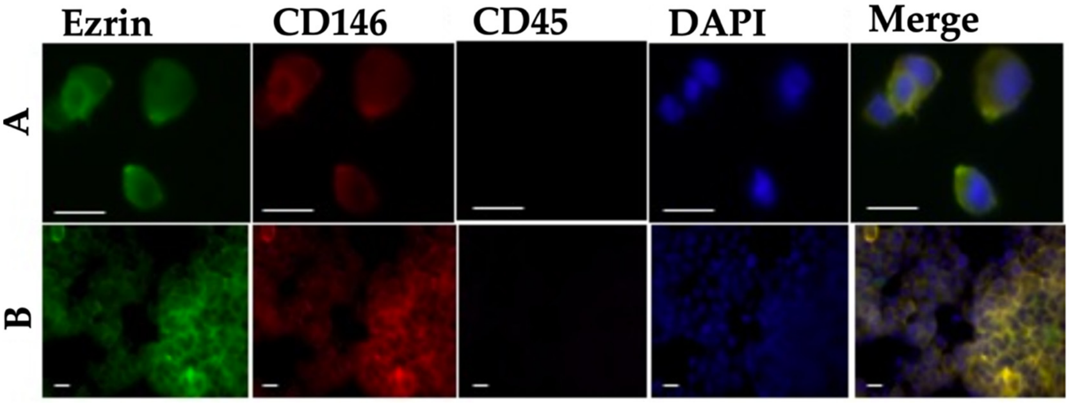

Supplementary Figure S2. Example of polarized and unpolarized HepG2 cells **Panel A)** Example of polarized HepG2 cells. **Panel B)** Example of unpolarized HepG2 cells. Anti-Ezrin-Alexa Fluor 488 staining (green), CD 146 (red), CD45/leukocytes (purple), DAPI/nuclear staining (blue) and merged images of all the fluorescence channels. Scale bar= 50 µm. 40x magnification.

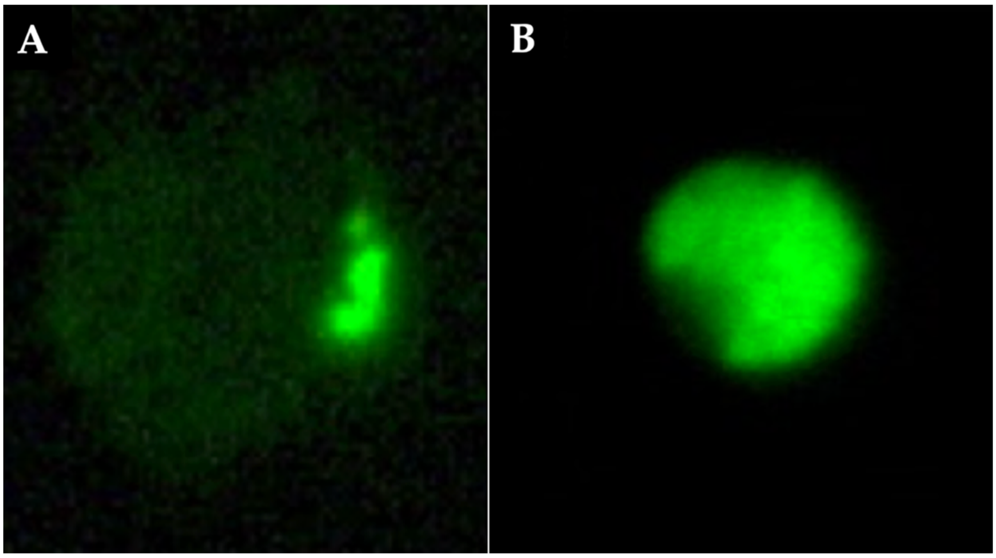

Supplementary Figure S3. Anti-Ezrin-Alexa Fluor 488 staining of polarized and unpolarized tumor cells ,**A)** Polarised tumor cell **B)** Unpolarised tumor cell. 40x magnification.

Clinical characteristics of 20 HCC and 18 NMLD patients		
	HCC cohort (n=20)	NMLD control cohort (n=18)
Age, years	Mean (range): 66.6 (50–82)<br>Median: 68	Mean (range): 64.8 (51–73)<br>Median: 67
Sex (M/F) (%)	12 (60%) / 8 (40%)	5 (27.8%) / 13 (72.2%)
Metastasis (%)	7 (35%)	0 (0%)
Alcohol abuse (%)	7 (35%)	1 (5.6%)
MASH (%)	5 (25%)	3 (16.7%)
Diabetes mellitus (%)	5 (25%)	6 (33.3%)
Hepatitis virus infection (%)	3 (15%)	0 (0%)
Liver cirrhosis (%)	11 (55%)	8 (44.4%)
MELD	0-10: 17	0-10: 12
	11-20: 2	11-20: 1
	21-30: 0	21-30: 4
	31-40: 1	31-40: 1
BCLC (%)	0=2 (10%)	n.A.
	A=7 (35%)	
	B=5 (25%)	
	C=6 (30%)	
Tumor size median (range) in cm	3.8 (0.7-14)	n.A.
Tumor size > 5 cm (%)	>5cm: 5 (25%)	n.A.
	≤ 5cm: 15 (75%)	n.A.
Preoperative therapy (%)	5 (25%)	3 (15%)

Operation Method (%)	Open: 14 (78%)	Open: 12 (86%)
	Laparoscopic: 4 (22%)	Laparoscopic: 2 (14%)
AFP, ng/ml	Negative (≤ 7.0): 10	n.A.
	Positive (> 7.0): 10	n.A.
Grading	G1: 2 (11%)	n.A.
	G2: 13 (72%)	n.A.
	G3: 3 (17%)	n.A.
Microvascular invasion (V)	No: 14 (78%)	n.A.
	Yes: 4 (22%)	n.A.
Tumor-free resection margin (R0)	No: 2 (11%)	n.A.
	Yes: 16 (89%)	n.A.
Recurrence	3 (17%)	n.A.
Mortality (%)	3 (15%)	3 (16.7%)
CTCs (%)	Positive: 19 (95%)	Positive: 2 (11.1%)
	Negative: 1 (5%)	Negative: 16 (88.9%)
Polarised CTCs (%)	Positive: 15 (75%)	Positive: 1 (5.6%)
	Negative: 5 (25%)	Negative: 17 (94.4%)

Supplementary Table S1. Demographics

Variable	*M*	*SD*	1	2	3	4	5	6	7	8	9	10	11	12	13
1. Sex	1.40	0.50													
2. Age	66.60	8.22	.46* [.02, .75]												
3. BCLC	2.75	1.02	-.00 [-.44, .44]	-.01 [-.45, .43]											
4. MELD Score	9.65	6.71	-.27 [-.64, .20]	-.41 [-.72, .03]	.16 [-.31, .56]										
5. Child Score	2.10	0.45	-.19 [-.58, .28]	-.48* [-.76, -.04]	.06 [-.39, .49]	.96** [.90, .98]									
6. Milan Criteria	0.10	0.31	-.27 [-.64, .19]	-.50* [-.77, -.08]	.25 [-.21, .62]	.60** [.22, .83]	.69** [.35, .87]								
7. Cirrhosis	0.55	0.51	-.29 [-.65, .18]	-.43 [-.74, .01]	.08 [-.38, .50]	.14 [-.33, .55]	.21 [-.26, .60]	.30 [-.16, .66]							
8. Alcohol Abuse	0.35	0.49	-.17 [-.57, .29]	-.33 [-.67, .13]	-.13 [-.54, .33]	.28 [-.19, .64]	.31 [-.15, .66]	.10 [-.35, .52]	.45* [.01, .75]						
9. Viral Hepatitis	0.15	0.37	-.06 [-.49, .40]	-.36 [-.69, .09]	-.18 [-.57, .29]	-.19 [-.58, .27]	-.10 [-.52, .36]	.33 [-.14, .67]	.38 [-.08, .70]	-.31 [-.66, .16]					
10. MASH	0.25	0.44	.00 [-.44, .44]	.14 [-.32, .55]	.15 [-.32, .55]	.01 [-.43, .45]	-.13 [-.54, .33]	-.19 [-.59, .27]	-.64** [-.84, -.27]	-.42 [-.73, .02]	-.24 [-.62, .22]				
11. Diabetes Mellitus	0.25	0.44	-.24 [-.61, .23]	-.26 [-.63, .21]	.15 [-.32, .55]	-.11 [-.53, .35]	-.13 [-.54, .33]	-.19 [-.59, .27]	.06 [-.39, .49]	.30 [-.16, .66]	-.24 [-.62, .22]	-.07 [-.49, .39]			
12. Metastasis	0.20	0.41	.10 [-.36, .52]	-.02 [-.46, .42]	.50* [.08, .77]	-.13 [-.54, .34]	-.11 [-.53, .35]	.25 [-.22, .62]	.20 [-.27, .59]	-.10 [-.52, .35]	.14 [-.32, .55]	-.29 [-.65, .18]	-.00 [-.44, .44]		
13. CTC	1.59	1.68	-.16 [-.56, .31]	-.20 [-.59, .26]	.20 [-.27, .59]	-.19 [-.58, .28]	-.17 [-.57, .30]	.32 [-.15, .67]	.38 [-.07, .71]	.05 [-.40, .48]	.43 [-.02, .73]	-.19 [-.59, .27]	-.02 [-.46, .43]	.15 [-.31, .56]	
14. p-CTC	0.56	0.70	-.31 [-.66, .15]	-.40 [-.72, .05]	.07 [-.38, .50]	-.17 [-.57, .29]	-.12 [-.53, .34]	.46* [.02, .75]	.36 [-.10, .69]	-.05 [-.48, .40]	.68** [.34, .86]	-.17 [-.57, .30]	.14 [-.33, .55]	.18 [-.29, .57]	.72** [.41, .88]

Supplementary Table S2. Means, standard deviations and correlations with confidence intervals in the HCC group. M and SD are used to represent mean and standard deviation, respectively. Values in square brackets indicate the 95% confidence interval for each correlation. The confidence interval is a plausible range of population correlations that could have caused the sample correlation. * indicates p<0.05. ** indicates p<0.01.
